# The treatment of large extraskeletal chondrosarcoma of the leg: Comparison of IMRT and conformal radiotherapy techniques^1^

**DOI:** 10.1120/jacmp.v2i1.2624

**Published:** 2001-01-01

**Authors:** Maria F. Chan, Chen‐Shou Chui, Karen Schupakm, Howard Amols, Chandra Burman, C. Clifton Ling

**Affiliations:** ^1^ Department of Medical Physics Memorial Sloan‐Kettering Cancer Center 1275 York Ave New York New York 10021

**Keywords:** chondrosarcoma, IMRT, 3D‐dose distribution, radiotherapy

## Abstract

Extraskeletal chondrosarcoma of the leg is a rare, malignant neoplasm with very few cases having been reported in the literature. In this study we investigate the possibility of using intensity modulated radiotherapy (IMRT) for this type of disease and demonstrate its advantages over conventional three‐dimensional (3D) conformal treatment. A case was presented of a patient with extraskeletal chondrosarcoma of the lateral compartment of the leg in which the target volume was 50 cm in length and twisted around the surrounding bones. Both the 3D conformal plan and IMRT plan were designed using the Memorial Sloan‐Kettering Cancer Center planning system. The IMRT plan produced a superior dose distribution to the patient as compared to the 3D conformal plan both in terms of dose conformity and homogeneity in the target volumes, and reduction of the maximum dose to the bone. The planning time of the IMRT plan was about 3–5 times shorter than that of the 3D conformal plan. It was demonstrated that the IMRT technique can be used not just for small tumors, but also for large and spiral‐shaped tumors close to critical organs. The IMRT method requires less planning time, and provides better target coverage with more sparing of critical structures. When planning patients with multiple target volumes receiving different prescribed doses, the IMRT technique can more easily meet this requirement.

PACS number(s): 87.53.–j, 87.90.+y

## INTRODUCTION

The majority of high‐grade soft‐tissue sarcomas surgically excised from the extremity require adjuvant radiation therapy as a component of their local management. High‐dose radiation, 6600 cGy, is the standard dose to the tumor bed in the setting of excision with negative margins. In the case of excision with microscopically positive margins, a dose of 7000 cGy is required. The radiation field is initially designed to extend 5–7 cm proximally and distally to the original tumor volume within the compartment of resection. This field is typically prescribed to a dose of 5000 cGy.^1^ ^–^ ^5^

It is not unusual to have a tumor, which measures 30 cm in length or more, in the proximal thigh, and therefore treatment fields are accordingly very long in these cases. It is technically challenging to treat such a volume homogeneously, while sparing at least one‐third of the circumference of the limb and half of the cortex of the adjacent bone. Complications of radiation therapy for extremity sarcomas include fibrosis, chronic edema, and decreased range of motion and increased risk of fracture.^6^ In extreme cases, amputation has been required due to profound fibrosis and severe normal tissue complications.^2^ ^,^ ^4^

High‐dose postoperative radiation therapy using proton beams for chondrosarcomas has been reported.^7^ ^–^ ^9^ Although it offers a good chance of lasting tumor control and survival, the proton facilities are not widely available. The use of an electron bolus for the superficial chondrosarcoma of the spine has been also reported,^10^ but to design a CT‐based 3D electron bolus is time consuming. Moreover, this method is not suitable for treating a large volume, such as the thigh. There are other techniques reported in the literature including radiosurgery and multiple field arc rotations for the treatment of chondrosarcoma.^11^ ^,^ ^12^

A conventional 3D treatment plan is difficult to design due to the variations in lesion shape after complete surgical excision. In particular, postoperative radiation therapy could not easily conform to tissue contour in a typical limb through a field over 30 cm in length. Neither could the muscle compartment be perfectly followed as it curves around the adjacent bone. In this paper we explore the possibility of IMRT and demonstrate its advantages over conventional conformal treatment for this difficult clinical application.

## METHODS AND MATERIALS

**Figure 1:**
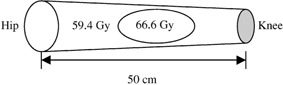
The target volume and the boost volume of the leg. The target volume is to receive 59.4 Gy and the boost volume 66.6 Gy.

A case was presented of a 44‐year‐old patient with extraskeletal chondrosarcoma of the lateral compartment of the leg in which the target volume was 50 cm in length and twisted around surrounding bones. The patient was positioned with the leg aligned along the longitudinal axis of the linear accelerator isocenter, and immobilized using a thermal plastic mold in a prone setup. After CT scanning, the physician outlined the target volume and prescribed the center region as a boost volume to receive a higher dose of 66.6 Gy while the rest of the target volume was to receive 59.4 Gy at a minimum, as shown in Fig. 1. The clinical criterion for the critical organ is 50% volume of the cortex of the bone should not receive more than 45 Gy.

**Figure 2:**
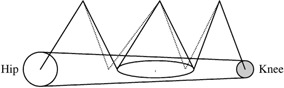
Field arrangements with three isocenters for the 3D conformal plan.

The 3D conformal plan and the IMRT plan were carried out on a planning system developed at the Memorial Sloan‐Kettering Cancer Center (MSKCC). The 3D conformal plan was designed with 18 MV photons using three isocenters with two opposed wedged pairs at each isocenter. The three isocenters were used to cover the entire length and provided more flexibility in beam weighting, which resulted in better dose homogeneity in the target volume. All field borders were matched at the plane of the isocenters and were shifted by 1 cm every other day in order to avoid any hot or cold spots near the field borders as shown in Fig. 2. No new 3D plan was generated to account for the 1‐cm shift of field borders because the dose distributions were expected to be almost the same as those in the original 3D plan.

The IMRT option in the planning system uses an iterative gradient algorithm to minimize an objective function which is represented as the sum of squares of the difference between the desired and the actual doses.^13^ The algorithm calculates the intensity distribution of each beam so that the resultant dose distribution from all beams best matches the one specified by the planner. The dose distribution from these intensity‐modulated beams is calculated with a pencil beam algorithm.^14^ The IMRT technique has been in clinical use at MSKCC to treat prostate,^15^ ^,^ ^16^ breast,^17^ and head and neck tumors.^18^ The dose distributions obtained from the IMRT plans are, in general, superior to those obtained from the conventional 3D conformal plans. It should be noted here that in some cases such as this, the target is close to the skin, hence part of the target is in the build‐up region. This could cause a potential problem in the inverse planning algorithm as it may result in unusually high intensities in certain parts of the beam in order to compensate for those points receiving low dose in the build‐up region. To circumvent this problem, in our inverse planning algorithm, there is an option to assign the dose in the build‐up region to the dose at $d_{\max}$. Of course, this change in dose occurs only in optimization. After the intensity distribution has been determined, the final dose calculation is still carried out the normal way, with the dose in the build‐up region calculated correctly.

For this patient, the IMRT plan was designed with the same energy, 18 MV, but with only a single isocenter at an extended source‐to‐skin distance (SSD). The reason for this is because for the IMRT plan, different weighting is already included as part of the intensity distribution for each beam, therefore there is no need to use multiple beams with different isocenters. But in order to cover the large area, extended SSD is needed. There were five fields with equally spaced gantry angles (from IEC standard 320° to 180° counterclockwise). These gantry angles were chosen to avoid irradiation of the contralateral leg. The intensity distributions were optimized by the optimization algorithm based on the prescribed dose to the three regions of tumor, including the higher dose to the boost volume, and the maximum dose constraint to the bone. Doses which deviated from the prescribed doses in the target or exceeded the constraints in the bone were subjected to penalties to the objective function. A total of approximately 8000 rays from all beams were used in the optimization. The spatial resolution of each ray was 0.2 cm in the leaf travel direction by 1 cm along the leaf width. The total number of points used in the optimization was approximately 15000. They were randomly selected in the target and critical organ structures. The total optimization time was about 1 minute.

## RESULTS AND DISCUSSION

**Figure 3:**
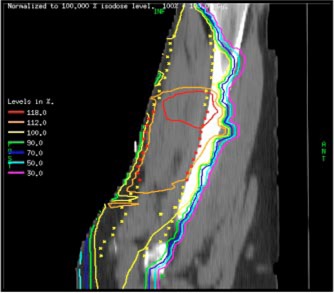
(Color) Sagittal plane dose distribution for the 3D conformal plan. The yellow crosses (×) indicate the target region where the prescribed dose is 59.4 Gy. The red crosses indicated the boost region where 66.6 Gy is desired.

The 3D and the IMRT plans were generated and the dose distributions and dose volume histograms (DVHs) were compared. Both the 3D and the IMRT plans met physician‐specified clinical criteria, namely, target dose and bone sparing. The dose distribution of the 3D conformal plan is shown in Fig. 3. The yellow crosses (×) indicate the target region where the prescribed dose is 59.4 Gy. The red crosses indicate the boost region where 66.6 Gy is desired. The displayed dose distribution was normalized to the prescribed dose, that is, 100% is equal to 59.4 Gy. The three dotted lines indicate the central axes of the three pairs of opposed fields. As can be seen in Fig. 3, most of the target is covered by the 100% isodose line, with a hot spot of 118% located in the inferior junction of the target and the boost regions. A significant portion of the bone also receives dose in excess of 100%. The 3D plan was also repeated using the 6 MV beams but the results are not shown because the dose homogeneity in the thick thigh region was not as good as that obtained with the 18 MV beams.

**Figure 4:**
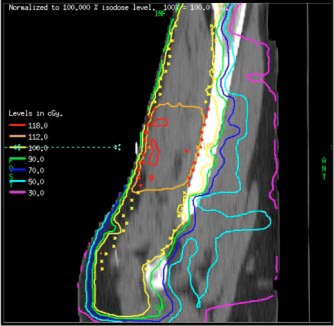
(Color) Sagittal plane dose distribution for the IMRT plan. The yellow crosses (×) indicate the target region where the prescribed dose is 59.4 Gy. The red crosses indicated the boost region where 66.6 Gy is desired.

**Figure 5:**
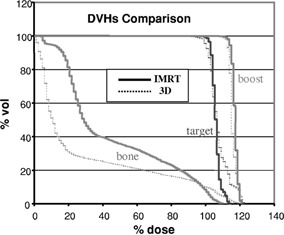
Comparison of DVHs of 3D conformal and IMRT plans.

The IMRT plan produced a superior dose distribution to the patient as compared to the 3D conformal plan in terms of both target conformity and bone sparing. The 100% and the 112% dose levels for the IMRT plan as shown in Fig. 4 conform better to the target and the boost volumes, respectively, than the 3D conformal plan shown in Fig. 3. For example, the volume receiving more than 112% dose in the target excluding the boost volume was less than 5% for the IMRT plan vs. 22% for the 3D plan. This is also seen in the comparison of dose‐volume histograms shown in Fig. 5, where superior conformity of the IMRT plan in the target volume is evident. In addition, the maximum dose to the bone, which is one of the clinical criteria, was reduced from 120% in the conformal plan to 108% in the IMRT plan. Although in the low‐dose region the IMRT plan irradiated more volume than the conformal plan, the dose delivered to 50% of the bone was 17 Gy, still well below the clinical criterion. All IMRT fields were determined automatically by the inverse planning system. As a result, the planning time was about 3–5 times shorter than that of the 3D conformal plan as the latter required many trial‐and‐error runs to determine the best choice of wedges and to adjust the beam weights. The IMRT plan also reduced hot and cold spots within the target volume, thereby eliminating the need for field abutments and feathering of the field junctions. It should be noted that in the IMRT plan, the volume of the normal soft tissue receiving 30–50% of the dose is more than that of the 3D plan. This is primarily due to the five beam directions used in the IMRT plan as opposed to the two tangential directions used in the 3D plan. Moreover, as the normal soft tissue is not declared as a critical organ, it is not considered by the inverse planning algorithm. The algorithm only tries to achieve the specified criteria, namely, uniform dose distribution to the boost and the target volumes, and reduction of the maximum dose to the bone.

In this study, we have planned but not actually treated the patient with the IMRT technique. The main reason was because this type of IMRT treatment requires large field size. Our current clinical version of the software does not have the feature of automatically splitting a large field into two or more smaller fields, with each field less than 14.5 cm wide, which is the limitation imposed by Varian's multileaf collimator (MLC). A research version of the software capable of splitting the field has been developed, but is still being tested. This version is expected to be available for clinical use soon.

## CONCLUSIONS

This example demonstrates that the IMRT technique can be used not just for small tumors, but also for large and spiral‐shaped tumors close to critical organs. The IMRT method is time saving, and provides better target coverage with considerable sparing of critical structures from high doses. In addition, when planning patients with multiple target volumes receiving different prescribed doses, the IMRT technique can more easily meet this requirement. The only apparent disadvantage of the IMRT plan for this particular patient would have been the increased daily set up time on the linear accelerator necessitated by the extended SSD. This planning study demonstrates a useful application of IMRT for a superior treatment for patients with a large chondrosarcoma of the leg.
